# Comparative Evaluation of Different Mint Species Based on Their In Vitro Antioxidant and Antibacterial Effect

**DOI:** 10.3390/plants14010105

**Published:** 2025-01-02

**Authors:** Ameni Sfaxi, Szilvia Tavaszi-Sárosi, Kovács Flórián, Katalin Patonay, Péter Radácsi, Ákos Juhász

**Affiliations:** 1 Department of Medicinal and Aromatic Plants, Institute of Horticultural Science, Hungarian University of Agriculture and Life Sciences (MATE), Villányi út. 29-43., H-1118 Budapest, Hungary; amenisfaxi0@gmail.com (A.S.); radacsi.peter@uni-mate.hu (P.R.); 2 Department of Agro-Environmental Studies, Hungarian University of Agriculture and Life Sciences, Villányi út. 29-43, H-1118 Budapest, Hungary; kovacs.florian@szte.hu; 3 Institute of Plant Sciences and Environmental Protection, Faculty of Agriculture, University of Szeged, Dugonics tér 13, H-6720 Szeged, Hungary; 4 Food and Wine Knowledge Centre, Eszterházy Károly Catholic University, Eszterházy tér 1., H-3300 Eger, Hungary; patonay.katalin@uni-eszterhazy.hu; 5 Department of Microbiology and Applied Biotechnology, Institute of Genetics and Biotechnology, Hungarian University of Agriculture and Life Sciences (MATE), Páter Károly utca 1., H-2100 Gödöllő, Hungary; juhasz.akos@uni-mate.hu

**Keywords:** *Mentha*, essential oil, antioxidant capacity, antibacterial activity

## Abstract

In our research six different mint species (peppermint, spearmint (five different chemotypes), Horse mint, mojito mint, apple mint (two different chemotypes), bergamot mint) have been evaluated by referring to their chemical (essential oil (EO) content and composition) and in vitro biological (antibacterial, antioxidant effect) characteristics. The EO amount of the analyzed mint populations varied between 1.99 and 3.61 mL/100 g d.w. Altogether, 98 volatile compounds have been detected in the oils. Antibacterial effects (inhibition zones, MIC, IC_50_ and MBC) were evaluated against *Escherichia coli*, *Salmonella enterica*, *Bacillus cereus* and *Staphylococcus aureus*. The best antibacterial effect was given by a carvacrol–thymol chemotype spearmint population (inhibition zone: 18.00–20.00 mm, MIC: 0.06 *v*/*v*%, IC_50_: 0.01–0.03 *v*/*v*%, MBC: 0.06, >2.00 *v*/*v*%). The least effective oil in the case of Gram-negative bacteria was bergamot mint (inhibition zone: 7.67–8.67 mm, MIC: 2.00, >2.00 *v*/*v*%, IC_50_: 0.11–0.25 *v*/*v*%, MBC: 2.00, >2.00 *v*/*v*%), while in the case of Gram-positive bacteria, oils containing dihydrocarvone as the main compound possessed the weakest antibacterial effect (inhibition zone: 9.00–10.00 mm, MIC: 1.00–2.00 *v*/*v*%, IC_50_: 0.22–0.37 *v*/*v*%, MBC: >2.00 *v*/*v*%). Interestingly, none of the oils could kill *B. cereus* in the applied concentrations.

## 1. Introduction

Mint species belong to the *Lamiaceae* plant family. The genus *Mentha* contains 18 species and 11 hybrids. The most well-known species is *Mentha × piperita* L. that is a hybrid of *Mentha aquatica* L. and *Mentha spicata* L. em. Huds. Probably *Mentha spicata* L. is also a hybrid of *Mentha suaveolens* L. and *Mentha longifolia* L. [1]. Dried leaves (*Menthae folium*) and the steam-distilled essential of peppermint *(Menthae piperitae aetheroleum)* and Japanese mint *(Menthae arvensis aetheroleum partim mentholum depletum*) are official drugs in the European Pharmacopoeia; the requirements of the drugs can be seen in the Hungarian Pharmacopoeia as well.

Mint species have been used for medicinal purposes for more than 2000 years owing to their different chemical compositions. They are rich in phenolic acids and flavonoids—especially rosmarinic acid, which has been intensively studied for their significant antioxidant and anti-inflammatory properties [2,3,4,5]. Studies have shown that the total phenolic content (TPC) in *Mentha* species is closely connected with their antioxidant ability; species like *Mentha spicata* and *Mentha suaveolens* show very high TPC and robust free radical scavenging action [6]. Moreover, several of these species are characterized by valuable essential oil content as well, which helps to explain their antibacterial qualities. For instance, similar main components include carvone, menthol, and menthone, which have been shown to have great antibacterial properties against common pathogens—*M. spicata*, *M. longifolia*, and *M. × villosa* [7]. *Mentha spicata*, for instance, has been particularly helpful against *Staphylococcus aureus* and *Escherichia coli*; *Mentha longifolia* has a strong antibacterial effect against *Bacillus cereus* and *Salmonella typhimurium* [8].

Among the abovementioned substances, menthol and menthol-based essential oils have demonstrated strong antibacterial effects against *E. coli* ATCC O157:H7 [9]. If the minimum bactericidal concentration of individual EO components is surveyed in the study of Soković and coworkers, a rank can be established like this: linalyl acetate = limonene = β – pinene < α-pinene < camphor < cineole = linalool << stretomycin < menthol = thymol < carvacrol.

The antimicrobial effects of mint species have long been known, and in recent decades, the antibacterial properties of mint EOs have been increasingly researched, because the excessive use of antibiotics and its negative consequences over the past two decades have led to increasingly stricter limitations on the use of antibiotics. For instance, concerns over the risks associated with antimicrobial agents resulted in the European Union banning the use of antibiotics as feed additives in 2006 [10]. Following this, numerous additional regulations have been implemented [11], including a target to reduce antibiotic use in human medicine by 20% by 2030 [12]. As the demand for antimicrobials remains, scientific efforts have increasingly focused on identifying viable alternatives, such as the exploration of bioactive compounds from plant extracts.

This chemical overlap implies that, despite their different habitats and shapes, *Mentha* species show a basic set of phytochemicals that support their antioxidant and antibacterial capabilities, therefore offering flexible options for both industrial and medical uses.

As we can see, many in vitro research data are available referring to the possible antimicrobial, antifungal, insecticidal, and allelopathic effects of mint oils [13,14,15,16,17], and the antioxidant properties of the plant extracts [6,18,19,20]. However, even if we have many literature data, their comparison is rather difficult because of the following reasons: different plant material, sampling mistakes (only one sample is collected without replications, sampling is not representative), non-adequate data on the plant material, different applied methods for the analysis, different bacteria and fungus lines, units, etc. The same conclusion has already been taken by Deans (2007) [21]; however, uniformly accepted sampling and analytical methods are still missing. Therefore, real comparable results can only be provided by complex studies, where several species are tested for several biological effects.

The repeatability of microbial methods also greatly depends on standardization; however, there are no specific standards applicable to essential oils, leading researchers to use significantly different methods, which complicates the comparability of the results obtained. For example, two authors reported very different MIC values in the case of a carvone-type *M. spicata* essential oil against *E. coli*: 0.005 µL/mL [22] and 12.5 mg/mL [23]. Besides methodological variations, researchers often concentrate on analyzing only one or a few plant extracts, and it is uncommon to find studies that test multiple plant species or multiple samples from each species. Until a universally accepted method for evaluating the antimicrobial effects of essential oils is established, the most reliable comparisons come from comprehensive studies that examine the antimicrobial activity of various essential oils against different microbes. Regarding mint species, the studies by Kowalczyk et al. [17] and Fazal et al. [24] can be regarded as a good basis to compare the possible antibacterial effect of different mint species.

Based on the abovementioned reasons our aim was to compare six different mint species (peppermint, spearmint (five different chemotypes), horse mint, mojito mint, apple mint (two different chemotypes), bergamot mint) referring to their chemical (essential oil (EO) content and composition) and in vitro biological (antibacterial, antioxidant effect) characteristics. A further aim was to reveal and understand the possible connection between the major and minor compounds and the tested biological effects in the mirror of the previous literature data.

## 2. Results

### 2.1. Phytochemical Characteristics of the Analyzed Mint Populations

The different mint populations were significantly separated based on antioxidant capacity (AC), total phenolic content (TPC), and essential oil content (EO) (F (20,174) = 154.65; *p* < 0.001; Wilks’ λ = 0.003; partial η^2^ = 0.94). The mint populations had a strong effect on all three variables: TPC (*p* < 0.001, partial η^2^ = 0.93), AC (*p* < 0.001, partial η^2^ = 0.94), and EO (*p* < 0.001, partial η^2^ = 0.83). In all three cases, the partial η^2^ values exceeded 83%, indicating that the differences between populations significantly influenced the variance in these variables.

**Figure 1:**
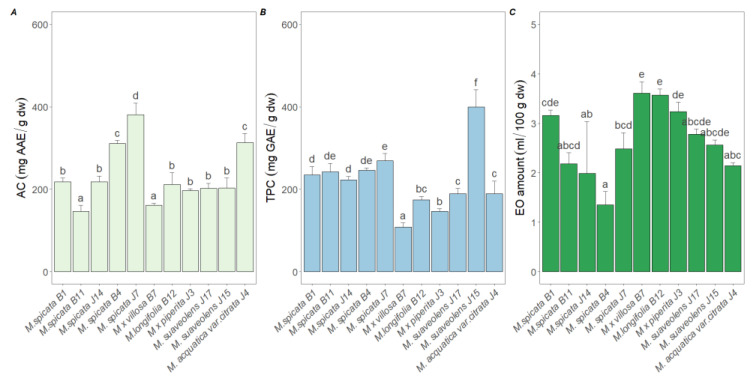
Comparison of antioxidant capacity (AC) (**A**), total phenolic content (TPC) (**B**), and essential oil (EO) amounts (**C**) across eleven *Mentha* (M.) populations. The results of the multiple comparisons of the MANOVA model are indicated by the letters above the graph. Statistical significance was determined using the Tukey post hoc test to compare the means of different groups after ANOVA with *p* < 0.05.

The highest antioxidant capacity value was observed in the *M. spicata* J7 population (381.04 ± 28.16 mg AAE/g d.w.), which was significantly higher than all other populations (Tukey test, *p* < 0.05). In contrast, the lowest AC values were measured in the *M. × villosa* B7 (160.89 ± 5.43 mg AAE/g d.w.) and *M. spicata* B11 (146.49 ± 14.72 mg GSE/g d.w.) populations, both showing significant differences compared to the highest value (Figure 1A).

The highest total phenolic content was recorded in the *M. suaveolens J15* population (399.63 ± 41.69 mg GAE/g d.w.), which was significantly different from the other populations. The lowest values were found in the *M. × villosa* B7 (107.91 ± 10.11 mg GAE/g d.w.) and *M. × piperita J3* populations (Figure 1B).

The highest essential oil content was found in the *M. × villosa* B7, *M. longifolia* B12, and *M. × piperita* J3 populations (3.61 ± 0.23 mL/100 g d.w.), which was significantly higher than all other populations. The lowest essential oil content was observed in the *M. spicata* B4 population (1.35 ± 0.27 mL/100 g d.w.) (Figure 1C).

**Figure 2:**
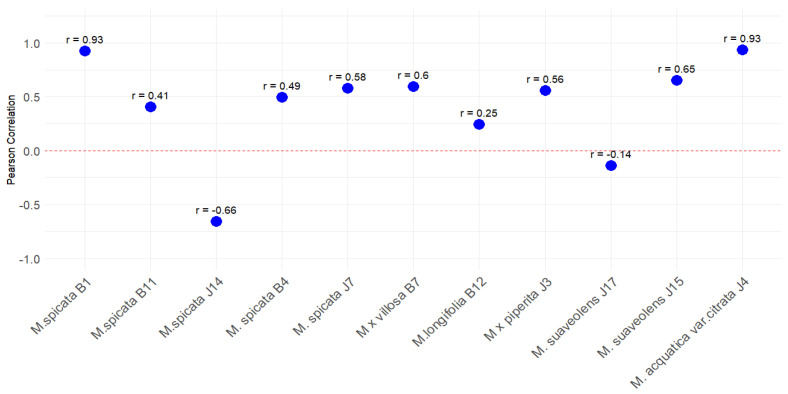
Pearson correlations between TPC and AC across different *Mentha* (M.) populations. (The plot shows the Pearson correlation coefficients (r) between total phenolic content (TPC) and antioxidant capacity (AC) across various mint species. Each blue dot represents the correlation coefficient for a specific species, labeled with its corresponding value (r). Positive values indicate a positive correlation between TPC and AC, while negative values indicate a negative correlation. The red dashed line at r = 0 represents no correlation.)

The results show significant variability in the strength and direction of the relationship between TPC and AC across different mint populations. In some cases, a strong positive correlation is observed, such as in *M. aquatica* J4 (r = 0.93) and *M. spicata* B1 (r = 0.93). These correlation coefficients suggest that in these species, higher levels of phenolic compounds are strongly associated with an increase in antioxidant capacity, indicating that phenolic compounds contribute significantly to antioxidant activity. In other populations, a moderate positive correlation is seen, such as in *M. × villosa* B7 (r = 0.60), suggesting that while the relationship exists, it may be influenced by other factors in addition to phenolic compounds. Interestingly, some species show weak or even negative correlations. *M. spicata* J14 exhibits a strong negative correlation (r = −0.66), indicating that an increase in phenolic content may reduce antioxidant capacity. In addition, *M. suaveolens* J17 shows a weak negative correlation (r = −0.14) (Figure 2). This phenomenon could be explained by the barrier of the FRAP method itself, since this method can measure the ferric reducing ability. Therefore, compounds with a lower redox potential than Fe^3+^/Fe^2+^ cause interferences [25]. For this reason, proper identification of the phenolic compounds (rosmarinic acid, caffeic acid, flavonoids, etc.) would be advisable in the future in the analyzed plant samples. 

### 2.2. Classification of Mentha Populations Based on Phytochemical Parameters

The mean of the relative percentage of essential oil components was calculated for all 11 mint populations. These averages reflect the unique chemical profiles of each population and highlight the specific compounds characteristic of each. In the *M. aquatica* J4 population, higher ratios were observed for linalool (23.12%) and linalyl-acetate (25.63%). This population also showed moderate amounts of neryl-acetate (12.98%) and 1,8-cineole (6.04%). The constitution based on open-chain monoterpenes (especially linalool and linalyl acetate) is typical of bergamot mints. However, the presence of neryl-acetate above 10% has not been reported from any *Mentha* taxon to date.

A unique essential oil composition was presented by *M. spicata* B11, where the main compounds were carvacrol (26.08%) 1,8-cineole (21.36%) and thymol (13.95%). *M. spicata* J14 exhibited high ratios of piperitenone oxide (55.67%) and 1,8-cineole (13.33%). All the other populations investigated here represent different branches of limonene 3-oxo and limonene-2-oxo pathways in wide diversity, as is usual in the genus *Mentha* [26]. The limonene-oxo products are often escorted by 1,8-cineole and completed by other cyclic monoterpenes. For example, in *M. suaveolens* J17 and *M. spicata* B1, among the miscellaneous compounds escorting limonene-oxo products, considerable amounts of *trans*-sabinene hydrate were found (9.91% and 10.29%, respectively). This compound represents the pathway of thymol action which is not considered to be typical in the genus *Mentha.*

Limonene-3-oxo ketones, alcohols and epoxides are dominant in mints J3, J15, B4, J17. *M. × piperita* J3 had high percentages of menthol (35.07%) and menthone (34.30%). Isomenthone (7.49%) and 1,8-cineole (5.18%) were also presented in moderate quantities. In the *M. spicata* B4 population, a high ratio of piperitenone oxide (56.28%) was observed. 1,8-cineole (15.50%) and β-myrcene (6.54%) were also prominent, distinguishing this population from others. Regarding *M. suaveolens* J17, *cis*-piperiton-epoxide (63.46%) and *trans*-sabinene-hydrate (9.91%) were notably present. Piperitenone oxide (5.45%) was also observed, while other compounds appeared in very low ratios. *M. suaveolens* J15 was characterized by exceptionally high concentrations of pulegone (87.51%). This population also contained neo-iso-pulegol (5.63%), with other compounds being present in minimal quantities.

Limonene-2-oxo ketones are prominent in mints J7, B7, B12, B1. The main essential oil compound of *M. spicata* J7 was *trans*-dihydrocarvone (36.99%) and *cis*-dihydrocarvone (32.79%). Other compounds, including β-myrcene and 1,8-cineole, were present in much lower amounts or were absent. *M. suaveolens* J15 was characterized by exceptionally high ratios of pulegone (87.51%). This population also contained neo-iso-pulegol (5.63%), with other compounds being present in minimal percentages. Regarding *M. suaveolens* J17, *cis*-piperiton-epoxide (63.46%) and *trans*-sabinene-hydrate (9.91%) were notably present. Piperitenone oxide (5.45%) was also observed, while other compounds appeared in very low ratios. *M. × villosa* B7 had high levels of L-carvone (64.61%) and limonene (18.81%). This population also contained moderate ratios of γ-terpinene (2.11%). In *M. longifolia* B12, high ratios of *cis*-dihydrocarvone (43.05%) and *trans*-dihydrocarvone (33.61%) were observed. The population also showed notable area percentages of β-caryophyllene (3.44%) and germacrene D (4.21%), as sesquiterpene compounds. *M. spicata* B1 was characterized by significant ratio of L-carvone (59.97%), *trans*-sabinene-hydrate (10.29%) and 1,8-cineole (9.28%). Rather unique essential oil composition was presented by *M. spicata* B11, where the main compounds were carvacrol (26.08%) 1,8-cineole (21.36%) and thymol (13.95%). *M. spicata* J14 exhibited high ratios of piperitenone oxide (55.67%) and 1,8-cineole (13.33%). Detailed results can be found in Table 1.

**Figure 3:**
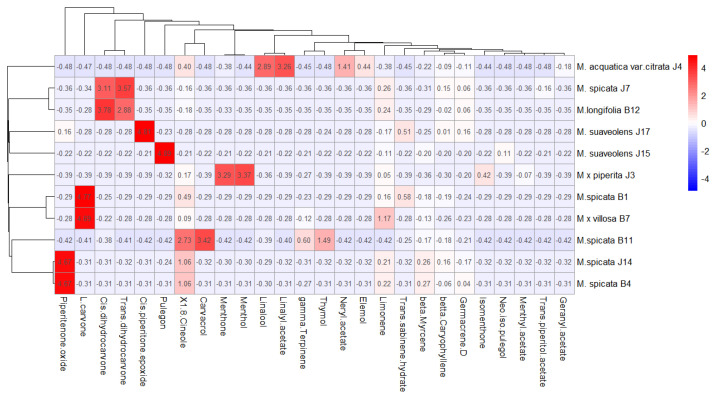
Heatmap of *Mentha* (M.) populations based on phytochemical components (Z-score normalized). The red color indicates a high Z-score, i.e., a deviation from the mean in the positive direction, while the blue color indicates a negative deviation. Values close to 0 (white) indicate that the concentration of the component is close to the mean, with no significant difference between populations. The lines represent hierarchical clustering, grouping populations and phytochemical components based on their similarity.

Following the analysis of the original phytochemical concentrations, the heatmap (Figure 3) presents the Z-score-normalized values of the essential oil components across various *Mentha* populations. The hierarchical clustering of these populations, based on their normalized phytochemical profiles, revealed clear groupings. *M. × villosa* B7 and *M. spicata* B1 formed a distinct cluster, primarily driven by elevated area percentages of piperitenone oxide and L-carvone, which differentiate them from other *Mentha* species.

*M. aquatica* J4 and *M. spicata* B1 showed the same Pearson correlation value, r = 0.93, which can be explained by the common present EO compound in each species, 1,8-cineole, with a very close amount 6.04% and 9.28%, respectively. The same rule applies to *M. × piperita* J3, *M. spicata* J7, and *M. × villosa* B7. These species showed a close Pearson correlation value—r = 0.56, r = 0.58, and r = 0.6, respectively. The major common EO compounds detected in these species are limonene with 4.12%, 5.89%, and 18.81% and 1,8-cineole with 5.18%, 1.91%, and 4.72%.

**Figure 4:**
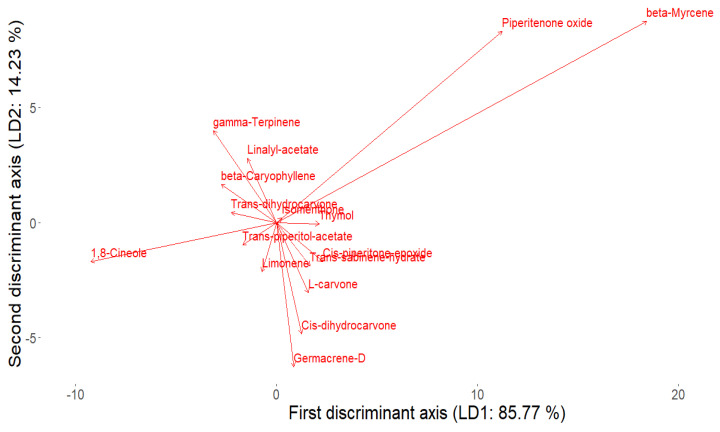
Discriminant analysis biplot of chemical components.

By analyzing the coefficients of the linear discriminants (LD1 and LD2), we determined which essential oil components contribute most to the separation of mint populations. As shown in Figure 4, β-myrcene was the most significant contributor to LD1 (coefficient = 18.43), while piperitenone oxide also had a strong effect on LD1 (coefficient = 11.22). These results, illustrated by the bar chart, highlight the essential oil components that most distinctly differentiate the mint populations.

**Figure 5:**
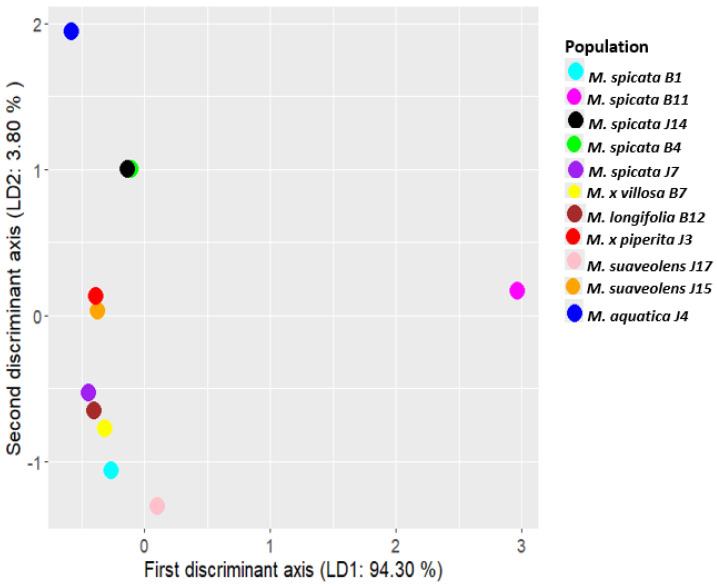
Scatter plot of *Mentha* (M.) populations based on the first and second discriminant functions (LD1 and LD2).

Figure 5 shows that each population is color-coded based on the corresponding point, as indicated in the legend. A distinctive grouping is observable on the basis of the biogenesis of the main essential oil components of the mints studied here. This is observable even in different populations of the same taxon. For example, *M. spicata* J14 (limonene-3-oxo epoxides, viz., piperitenone oxide, escorted by cineole) and *M. spicata* B11 (cymene compounds: thymol, carvacrol) are separated along the LD1 axis. Surveying the whole LD1xLD2 plane, three major groups are visible. One of them consists solely of the cymene-type *M. spicata* B11. The second is also a sole sample: bergamot mint J4, characterized by a high value of LD2. The separation may be a consequence of its unique EO chemistry based on linalyl acetate/linalool/neryl-acetate (open-chain monoterpenes) and 1,8-cineole. The third group of points (EO samples) are organized from batches rich in different limonene-oxo derivatives, being typical in the genus *Mentha*. This group has multiple subgroups, representing the different branches of the limonene-2-oxo pathway (leading to ketones of carvone class) along with the limonene-3-oxo pathway (ketones, alcohols and epoxides in the menthone class), completed by various non-limonene-derived terpenoids (e.g., 1,8-cineole, t-sabinene hydrate, and myrcene).

The abovementioned *M. spicata* EO (J14) bears practically the same coordinates in the LD1xLD2 plane as *M. spicata* B4, representing highly similar ratios of piperitenone epoxide (subclass: limonene-3-oxo epoxides) and 1,8-cineole. These two build a subgroup in the third group of points, bearing high values of LD2. EOs of *Mentha x piperita* J3, *Mentha suaveolens* J15 provide the next subgroup. J3’s and J15’s EOs are based on limonene-3-oxo ketones and alcohols. These are menthone in J3 along with the leading compound menthol; and pulegone escorted by neo-isopulegol in J15. Approximately the origo with gradually decreasing value of LD2 but increasing in LD1, the subgroups consisting of EOs based on limonene-2-oxo ketones can be seen. *M. spicata* J7 and *M. longifolia* B12 are both based on dihydrocarvone isomers which bring them into close proximity in LD1xLD2 plane. Interestingly, *M. × villosa* B7 (L-carvone + limonene) falls closer to them than the other carvone-type mint oils (*M. spicata* B1, *M. suaveolens* J17). In addition, *M. spicata* B1 (L-carvone/t-sabinene hydrate/cineole) made a separate subgroup.

### 2.3. Evaluation of Antibacterial Activity

As the first step in investigating the antimicrobial effect, we determined the inhibition zones of the essential oils against all four bacteria using the disc diffusion technique. In the case of the two Gram-negative bacteria, only *M. spicata* B11, which contains carvacrol and thymol, exhibited a significant inhibition zone (greater than 20.00 mm), while for the other ten cases, the diameters of the inhibition zones were found to be 7.00–12.00 mm. For the Gram-positive bacteria, *M. spicata* B11 also showed the strongest inhibition, but the EO of *M. aquatica* J4 demonstrated a similar level of inhibition (20.67–21.00 mm). Interestingly, in all other tests (MIC, IC_50_, and MBC), the EO of *M. aquatica* J4 did not show significant antimicrobial activity against the Gram-positive bacteria either, although it was slightly more effective than against the Gram-negative bacteria. In the case of *B. cereus*, larger inhibition zones were measured for *M. spicata* J14, *M. spicata* B4, and *M. suaveolens* J17 (14.33, 14.33 and 14.00 mm, respectively). For *S. aureus*, the essential oils of *M. × villosa* B7 (18.67 mm), *M. spicata* B1 (21.67 mm), and *M. × piperita* J3 (16.33 mm) exhibited greater inhibition zones. The inhibition zone (and MIC) values of the antibiotic used as a reference met the CLSI standards [27], thereby validating our results. The diameters of inhibition zones and all other relevant data of the antibacterial activity test are presented in Table 2.

The MIC values of EOs for *E. coli* were in the range of 0.06–2.00% *v*/*v*. The EO from carvacrol and thymol containing *M. spicata* B11 showed the strongest (MIC = 0.06% *v*/*v*) effect not only for this bacterium, but also for all other bacteria and all parameters tested. The EO from *M. aquatica* J4 (main compounds: linalool and linalyl-acetate) showed the weakest effect (MIC = 2.00% *v*/*v*). The MIC value of most essential oils was found to be 0.50% *v*/*v*, with the exceptions of *M. × villosa* B7 (MIC = 0.25% *v*/*v*) and *M. spicata* B4 (MIC = 1.00% *v*/*v*). In the case of the other Gram-negative bacterium (*S. enterica*), the MIC values were similarly distributed: *M. spicata* B11 was the most effective (MIC = 0.06% *v*/*v*), while *M. aquatica* J4 showed the weakest effect. The EO of *M. aquatica* J4 did not exhibit complete inhibitory activity even at the highest concentration tested (MIC > 2% *v*/*v*). For six essential oils, the MIC was 0.50% *v*/*v*, while for three it was 1.00% *v*/*v*. The higher MIC values were primarily associated with essential oils containing dihydrocarvone (from *M. longifolia* B12 and *M. spicata* J7). The *M. spicata* B4 essential oil, containing piperitenone oxide and 1,8-cineole, showed a MIC value of 1.00% *v*/*v* against both Gram-negative bacteria, which indicates a weaker antibacterial effect compared to the nearly identical EO of *M. spicata* J14 (MIC = 0.50% *v*/*v*). This observation was supported by the IC_50_ and MBC values not only for the Gram-negative bacteria, but also for the Gram-positive bacteria.

The MIC values of EOs for *S. aureus* were in the range of 0.06–1.00% *v*/*v*, while the MIC values for *B. cereus* ranged from 0.06 to 2.00% *v*/*v*. For both bacteria, *M. spicata* B11 exhibited the strongest effect (MIC = 0.06% *v*/*v*), while the essential oils containing dihydrocarvone from *M. longifolia* B12 (MIC = 1.00 and 2.00% *v*/*v*) and *M. spicata* J7 (MIC = 1.00% *v*/*v*) showed the weakest antimicrobial activity. The essential oil of *M. × piperita* J3, which contains menthol and menthone, exhibited the second strongest antibacterial effect (MIC = 0.13% *v*/*v*), but the oil of *M. suaveolens* J17, containing *cis*-piperitone-epoxide, also proved similarly effective (MIC = 0.13% and 0.25% *v*/*v*). In the case of *B. cereus*, one essential oil had a MIC value of 0.25% *v*/*v* (*M. spicata* J14, piperitenone oxide), while five had a MIC of 0.50% *v*/*v* (essential oils containing carvone, pulegone, piperitenone oxide, and linalool/linalyl acetate). For *S. aureus*, along with *M. spicata* J14, *M. suaveolens* J15 also had a MIC of 0.25% *v*/*v*, while for four other essential oils, the MIC was found to be 0.50% *v*/*v*.

When examining the MBC values, we can conclude that none of the essential oils were able to kill *B. cereus* even at a concentration of 2.00% *v*/*v*. Although the growth of this bacterium was effectively inhibited by several essential oils, likely due to its endospore-forming ability, the EOs were unable to kill *B. cereus*. For *S. aureus*, *M. spicata* B11 had the lowest MBC value (0.06% *v*/*v*), which was identical to the MIC value. The MBC value of *Mentha x piperita* J3 was found to be 0.50% (slightly higher than MIC value of 0.13%). Besides these, only the EOs containing piperitenone oxide and *cis*-piperitone-epoxide were able to kill *S. aureus*, though only at higher concentrations of 1.00% and 2.00%.

The MBC value for all other EOs was found to be >2.00% *v*/*v*. For the two Gram-negative bacteria, the MBC values were much closer to the MIC values in most cases compared to the Gram-positive bacteria. In the case of *E. coli*, with the exception of *M. × villosa* B7 (MBC = 0.50% *v*/*v*, MIC = 0.25% *v*/*v*), the MIC and MBC values were identical for all essential oils, suggesting that the inhibitory concentration also kills the cells. For *S. enterica*, the MBC was higher than the MIC in two cases (*M. spicata* B4 and *M. × piperita* J3), while in all other cases, the two values were found to be identical.

## 3. Discussion

The outcomes of this study on *Mentha* populations match with similar studies highlighting the variation in antioxidant capacity (AC), total phenolic content (TPC), and essential oil (EO) relative percentage over many species and environmental situations. Another research noted significant AC values in *M. spicata* populations due to high rosmarinic acid content, and the high antioxidant capacity of *M. spicata* J7 population demonstrates a significant concentration of phenolic compounds. Conversely, the lower AC values recorded in *M. × villosa* B7 and *M. spicata* B11 populations (160.89 ± 5.43 mg AAE/g d.w and 146.49 ± 14.72 mg AAE/g d.w, respectively) line up with prior research showing variability based on genetic variations and growth conditions.

The *M. suaveolens* J15 population’s observed higher TPC reflects earlier research linking high phenolic content to higher antioxidant capacity. A similar study [7] on *Mentha* species with greater TPC revealed increased antioxidant activity, therefore verifying the relationship between TPC and AC throughout population. The much lower TPC in *M. × villosa* B7 and *M. × piperita J3* populations, and the great difference between the two *M. suaveolens* populations, supports even further the idea that phenolic content can vary widely, even inter- and intraspecific.

It is the correlation between antioxidant capacity (AC) and total phenolic content (TPC) among many *Mentha* species that explains what is going on in the background. Results from many additional investigations line up with the high positive associations reported in *M. aquatica* J4 (r = 0.93) and *M. spicata* B1 (r = 0.93). For instance, a study on several *Mentha* species revealed strong links between TPC and AC, underlining the importance of phenolic components such as rosmarinic acid and eriocitrin in antioxidant activity [6].

On the other hand, the modest correlation in *M. × villosa* B7 (r = 0.60) reflects results of some researchers [28], whereby environmental variables and genetic elements were discovered to affect the strength of the association between TPC and AC. This implies that other molecules such as flavonoids or environmental stressors may potentially be involved even if phenolics add to antioxidant action.

Though less often than very unusual, the negative associations seen in *M. spicata* J14 (r = −0.66) and *M. suaveolens* J17 (r = −0.14). Increased TPC does not always result in better antioxidant capacity in some populations of *Mentha*, according to a 2020 study, which is most likely due to the presence of antagonistic chemicals or phenolic degradation. This suggests a more complicated interaction between TPC and AC in some populations that might possibly be altered by environmental conditions, timing of harvest, or extraction technique.

Another potential cause of the unconventional correlation between TPC and AC may be an untypical flavonoid profile. If the mint plant is rich in apigenin or kaempferol derivatives and/or hesperetin glycosides instead of eriocitrin (eriodyctiol-7-O-rutinoside), luteolin glycosides or quercetin glycosides, it may result in lowered AC, especially if it associates with low caffetannin production. In horsemint, an apigenin/kaempferol based batch is already described previously from Serbia [29]. Hesperetin, apigenin and kaempferol do not bear the free catechol moiety, viz., free OH groups at C3′, C4′ positions at the isolated (B) ring of the flavonoid backbone, while eriodyctiol, luteolin and quercetin derivatives possess it. This structural unit has key importance in antioxidant (and chelator) efficiency of flavonoids [26,28]. To Fe (III)/(II) chelator properties and anti-rancidity effect of eriocitrin, see the study of Yao et al. 2022 [5]. To answer if the flavonoid profile of J14 contributes to the unusual correlation of TPC and AC, it is necessary to determine polyphenol composition in the plant samples investigated here by applying HPLC/LC-MS method. We have already started these investigations.

With regard to essential oil amount, *M. × villosa* B7, *M. longifolia* B12, and *M. × piperita* J3 populations (3.61 ± 0.23 mL/100 g d.w) have more EO than *M. spicata* B4 (1.35 ± 0.27 mL/100 g d.w.). A study [21] revealed that EO content in *Mentha* species fluctuates greatly depending on both genetic elements and environmental stresses such light and water resources fit this discrepancy. The varied chemical makeup of *M. longifolia* and *M. × piperita* has been connected to their high EO levels; especially, the presence of menthol and carvone, which are main causes of their bioactive characteristics.

As seen by the variations in important chemicals like linalool, menthol, and 1,8-cineole, the chemical makeup of *Mentha* species differs greatly throughout populations. These results fit current studies on the variation in essential oil content in *Mentha* populations. For example, research demonstrating that linalool greatly influences the aromatic profile and possible antibacterial activities of different *Mentha* species [26] are in line with the high linalool content (23.12%) identified in *M. aquatica* J4. As noted in other essential oil-bearing plants, this chemical is also well known for reducing stress [30]. Linalool and linalyl acetate are typical constituents in bergamot mint EOs, both in *M. aquatica* var. *citrata* and *M*. *× piperita* var. *citrata*. However, the high percentage of neryl-acetate is untypical. To date, no similar data have been found. Geranyl or neryl derivatives as main components are rarely reported in the genus *Mentha*. In an Israeli study [31], glandular trichomes of *M*. *aquatica* var. *citrata* leaves synthesized neryl and geranyl acetates both on field and in vitro, but deeply under the concentrations observed in our work. The Israeli authors consider these volatiles as some byproduct of linalool synthesis. Throughout literature of the genus, only a few cases on geranyl/neryl derivatives as major constituents (5% +) are reported. One is a Croatian batch of *M. citrata* (*M. viridis × M. aquatica*) [32]. The EO contained linalyl acetate (21.46%), linalool (13.68%), 1,8-cineole (12.51%), geranyl acetate (8.66%), β-myrcene (8.10%), α-terpineole (7.38%) *cis*-β-ocimene (5.27%) completed by neryl-acetate and free geraniol (3.56% and 3.18%, respectively.) Another is not bergamot mint but a Moroccan wild-growing *M. suaveolens* ssp. *timija* population bearing geraniol (12.6%) among miscellaneous volatiles as piperitenone oxide (58%) and a fenchane-backbone alcohol (9.8%) [33]. This latter contained neither linalool nor linalyl acetate.

The known menthol-rich profile of *M. × piperita*, which is extensively consumed for its cooling and analgesic qualities, supports the higher menthol (35.07%) in *M. × piperita* J3 [34]. Studies on the extensive therapeutic usage of this species confirm the strong scent and medicinal potential of other chemicals like isomenthone (7.49%), which underlines even more their presence.

Spearmint showed the greatest essential oil variability among the investigated mint species, where L-carvone, dihydrocarvones, piperitenone oxide, and surprisingly, carvacrol/1,8-cineol were detected as main EO compounds in the different populations. These results are in accordance with the previous conclusions of [35,36,37,38], where the authors emphasized the great genetic diversity of this species, especially in the wild-growing populations. Most cultivated varieties are of the L-carvone type [39,40]. For the presence of dihydrocarvone, we cannot find any previous literature data—Sivropoulu et al., 1995 [41] described dihydrocarveole as the main compound (38.80%). Piperitenone oxide has been described as the second main compound in the EO of spearmint in some research [42] with the ratios of 15.68 and 26.20%. Only in two cases, 1,8-cineol was given as the man essential oil compound [43]. Referring to the carvacrol, it is important to note, that cymene compounds are atypical in the genus *Mentha*. However, in some cases, their presence was confirmed—in spearmint carvacrol was detected in 49.60% [44]; in horsemint thymol was detected in 13.30% [45], and in 18.60% [4]; and one time carvacrol was the main compound with 20.56% [46].

Conversely, the occurrence of pulegone in *M. suaveolens* J15 (87.51%) is noteworthy, as various studies [47] reveal that pulegone is a monoterpene showing strong insecticidal and antibacterial activities. It is important to note that, although this ketone may be useful as an antimicrobial and insecticidal agent, it is severely toxic if consumed (causing hepatic injuries, and miscarriage in women).

As noted in recent studies on the pharmacological features of *Mentha* species, high levels of *trans*-dihydrocarvone (43.05%) in *M. longifolia* B12 and *cis*-dihydrocarvone (32.63%) in *M. spicata* populations reflect the unique aromatic and possible therapeutic effects these compounds bring.

The diversity in essential oil content and composition among various *Mentha* species highlights the intricate chemical ecology of the genus affected by environmental variables and genetic elements. More comparative research would help to properly investigate the pharmacological possibilities and industrial uses of these several profiles.

Our aim was to conduct a detailed investigation of the antibacterial activity of EOs from six mint species (a total of 11 chemotypes). In addition to determining inhibition zones using the commonly applied disc diffusion method and MIC values through microdilution, we also assessed the IC_50_ and MBC values of the EOs against two Gram-positive and two Gram-negative bacteria. The selection of samples included plants containing the main essential oil components typically characteristic of mints (e.g., menthol/menthone, piperitenone oxide, *cis*- and *trans*-dihydrocarvone, and l-carvone), as well as those with components less commonly found in mint species (e.g., linalool/linalyl acetate, thymol). Our objective was to compare the antibacterial effects of the essential oils with precision, employing various techniques within the same experimental framework. Across all four bacterial strains and testing methods, the *M. spicata* B11 thymol-containing EO (widely known for its strong antibacterial effect) proved to be the most effective. The MIC values were identical for all bacterial strains (0.06% *v*/*v*), and the agar diffusion method revealed similar inhibition zones of approximately 20.00–22.33 mm. However, IC_50_ comparisons indicated that Gram-positive bacteria were slightly more sensitive (IC_50_ for *B. cereus*: 0.01, *S. aureus*: 0.02) than Gram-negative bacteria (IC_50_ for *E. coli*: 0.03, *S. enteritidis*: 0.03) to this EO. The MBC values were identical to the MIC values for all strains except *B. cereus*. In the case of *B. cereus*, none of the EOs were able to kill it at the maximum tested concentration (2.00% *v*/*v*), likely due to the ability to form endospores. However, in other studies, *B. subtilis* showed identical MIC (0.13% *v*/*v*) and MBC (0.13% *v*/*v*) values when treated with *M. suaveolens* EO [20]. Other authors were unable to determine MIC or MBC values for *B. subtilis* when using *M. pulegium* essential oil, even at their maximum tested concentration (2 mg/mL) [48].

In the case of the two Gram-positive bacteria, the essential oil of *M. × piperita* J3, containing menthol/menthone, demonstrated the second highest efficacy based on the MIC values (both 0.13% *v*/*v*). For *S. aureus*, it was also the second most effective in terms of MBC (0.50% *v*/*v*). Based on IC_50_ values, the results for *S. aureus* were similar to those observed with essential oils containing L-carvone, *cis*-piperitone-epoxide, and pulegone (IC_50_ 0.06–0.09). A similar trend was observed with *B. cereus*, although the IC_50_ values for L-carvone-containing oils were higher (0.15 and 0.19). Regarding the two Gram-negative bacteria, the essential oil of *M. × piperita* J3 ranked second in efficacy among the tested compounds based on IC_50_ values. However, with MIC values, several other essential oils exhibited similar efficacy. Notably, for *E. coli*, *M. × villosa* B7 (L-carvone type) proved to be more effective in terms of MIC (0.25% *v*/*v*) than the menthol-containing oil (MIC 0.50% *v*/*v*). For *S. enterica*, the MBC value was higher (1.00% *v*/*v*) than that of the essential oils containing L-carvone, *cis*-piperitone-epoxide, and pulegone (MBC = 0.50% *v*/*v*). Interestingly, *M. × piperita* J3 essential oil exhibited a significant inhibition zone (16.33 mm) only against *S. aureus*, while the other three bacteria displayed inhibition zones of around 10 mm.

The L-carvone-containing essential oils (*M. × villosa* B7 and *M. spicata* B1), *cis*-piperitone-epoxide-containing *M. suaveolens* J17, pulegone-containing *M. suaveolens* J15, and one piperitenone oxide-containing EO (*M. spicata* J14) exhibited similar antimicrobial activity. Based on MIC values, the L-carvone-containing essential oils appeared slightly more effective against Gram-negative bacteria, whereas the others (*M. suaveolens* J17, *M. suaveolens* J15, *M. spicata* J14) inhibited Gram-positive bacteria more effectively. However, based on IC_50_ values, *S. aureus* was the most sensitive to L-carvone-containing EOs. In the case of MBC, these EOs were more effective at killing Gram-negative bacteria than Gram-positive ones.

The two piperitenone oxide-type EOs, *M. spicata* J14 and *M. spicata* B4, exhibited identical performance in agar diffusion tests, with only *B. cereus* showing a significantly larger inhibition zone (14.33 mm) for both EOs. For all other bacteria, both EOs had inhibition zones of 9.00–10.33 mm. Nevertheless, based on MIC, IC_50_, and MBC, the EO of *M. spicata* J14 exhibited a much stronger antibacterial effect compared to *M. spicata* B4, despite their nearly identical chemical composition. It is likely that compounds not commonly identified in essential oil analysis might influence the antimicrobial efficacy of these two oils. Similar observations, where rather similar EOs possess varying antimicrobial activities, have been reported by others [17]. However, both piperitenone oxide-containing essential oils were more effective against Gram-positive bacteria than Gram-negative ones, except in the case of MBC.

The main active compounds of the EOs of *M. longifolia* B12 and *M. spicata* J7 were *cis*-dihydrocarvone and *trans*-dihydrocarvone. In the agar diffusion test, they formed only minimal inhibition zones for all bacteria; however, based on MIC, IC_50_, and MBC values, they are more effective against Gram-negative bacteria than against Gram-positive bacteria. The EO of *M. aquatica* J4, containing linalool and linalyl acetate, was the least effective against Gram-negative bacteria but demonstrated significant inhibition against Gram-positive bacteria using the agar diffusion technique (20.67 and 21.00 mm). Based on MIC values, it was similarly effective as the L-carvone-EOs. Moreover, for *B. cereus*, it was the fourth most effective oil based on IC_50_ among the 11 EOs tested.

Based on our results, it can be concluded that, in terms of antimicrobial efficacy, the *M. spicata* B11 thymol-type EO proved to be the most effective overall. In most cases, it was followed in effectiveness by the *M. × piperita* J3 EO, which contains menthol and menthone. EOs rich in l-carvone, *cis*-piperitone-epoxide, pulegone, and piperitenone oxide exhibited similar antibacterial properties, although the intensity of their effects varied slightly depending on the bacterial species and between Gram-positive and Gram-negative groups. EOs containing dihydrocarvone or linalool/linalyl acetate were the least effective when analyzing the data across all four bacterial strains. However, when grouped by cell wall type, dihydrocarvone-type EOs showed greater inhibition of Gram-negative bacteria, while those with linalool were more effective against Gram-positive bacteria.

It is a widely accepted notion that EOs from mint species are generally less effective against Gram-negative bacteria due to their distinct cell wall structure. Unlike the single membrane of Gram-positive bacteria, the cell wall of Gram-negative bacteria serves as a biological barrier to antibacterial agents [49]. Our investigations support this observation, particularly in the case of the commonly studied menthol-containing *M. × piperita* essential oil, as well as with EOs rich in *cis*-piperitone-epoxide, piperitenone oxide, dihydrocarvone, and linalool, which inhibited Gram-positive bacteria more effectively than Gram-negative bacteria. Conversely, EOs containing L-carvone and pulegone showed greater inhibition of Gram-negative bacteria. However, it should be noted that it is difficult to draw definitive conclusions regarding bacterial groups with distinct cell wall types based on data from only two bacterial species, even if similar findings have been observed by other researchers [17]. To truly understand the effects of different EOs on Gram-positive and Gram-negative bacteria, it would be necessary to test these compounds on a larger number of bacterial strains using standardized microbiological methods.

## 4. Materials and Methods

### 4.1. Plant Materials

The basic plant material for the studies were collected from the experimental field of the Department of Medicinal and Aromatic Plants, MATE University. Standing cultures were made by vegetative propagation of the mother plants in 2022. For our investigations, the following populations have been chosen:

*Mentha spicata*—B1, B11, J14, B4, J7; *Mentha × villosa*—B7, *Mentha longifolia*—B12, *Mentha × piperita*—J3; *Mentha suaveolens*—J17, J15; *Mentha aquatica* var. *citrata*—J4. Codes refer to the population names in our gene bank.

Sample collection (200 g of fresh material) was performed in 2023, at the end of July. The 40 cm long flowering stems were cut, then dried in a natural way, in shade for 3 weeks. Dried leaves were removed from the stem and were used for further analysis.

### 4.2. Essential Oil Extraction

The plant samples (10 g, in three replications) underwent hydro-distillation (by using 500 mL water for a duration of 2.5 h) using a Clevenger-type apparatus, following the protocol outlined in the VII. Hungarian Pharmacopoeia. After collecting the oils, the remaining water was taken out by using anhydrous sodium sulphate. All essential oil samples were stored in tightly sealed vials within a refrigerator at a temperature of 4 °C until the time of analysis.

### 4.3. Gas Chromatographic–Mass Spectrometric Analysis

The GC–MS analyses were carried out on each EO sample using an Agilent Technologies 6890N instrument equipped with HP–5MS capillary column (30 m × 0.25 mm i.d. × 0.25 μm) and an Agilent Technologies MS 5975 inert mass selective detector. The temperature program was the following: initial temperature 60 °C, then increased by a rate of 3 °C/min up to 240 °C; the final temperature was maintained for 5 min. The carrier gas was helium (1 mL/min); injector and detector temperatures were 250 °C. Split ratio: 30:1. A volume of 10 µL of EO was diluted by n-hexane to 1 mL and from this, the injected quantity was 0.2 µL.

The identification of the constituents was based on the comparison of the retention times with those of authentic samples, comparing the linear retention indices relative to a series of *n*-hydrocarbons (C_9_–C_23_) using the generalized equation of Van den Dool and Kratz (1963) [50], and by using commercial databases (NIST and Wiley) for the mass spectra analysis.

### 4.4. Antioxidant Properties

#### 4.4.1. Antioxidant Capacity (AC)

The ferric reducing antioxidant power (FRAP) assay was performed according to the method described by Benzie and Strain (1996) [51]. An amount of 1 g dried and powdered plant material was extracted by 100 mL hot distilled water then filtered after 24 h. All measurements were performed in triplicate. Ferric reducing antioxidant power (FRAP) reagent was prepared freshly to contain sodium acetate buffer (pH 3.6), TPTZ (2,4,6-tripiridil-s-triazin) in HCl and FeCl_3_×6H_2_O solution (20 mmol/L), in proportion 10:1:1(*v*/*v*/*v*), respectively. A volume of 10 µL of sample was added to 1.5 mL of FRAP reagent and 40 µL distilled water. The absorbance was measured at 593 nm after 5 min using in a Thermo Evolution 201 spectrophotometer (Thermo Fisher Scientific, Waltham, MA, USA). Blank was prepared to contain distilled water instead of extract. FRAP values of samples were calculated from standard curve equation and expressed as ascorbic acid equivalent/g dry weight extract (AAE/g dw.).

#### 4.4.2. Total Phenol Content (TPC)

The total phenolic content was determined by the modified method of Singleton and Rossi (1965) [52]. An amount of 1 g dried and powdered plant material was extracted by 100 mL hot distilled water then filtered after 24 h. All measurements were performed in triplicate. A 0.5 mL sample was taken into a test tube, then 2.5 mL Folin–Ciocalteau’s reagent (10 *v*/*v*%) was added. After incubation for 1 min, 2 mL of sodium carbonate (0.7 M) was added. The absorbance was measured at 760 nm in a Thermo Evolution 201 spectrophotometer (Thermo Fisher Scientific, Waltham, MA, USA) after incubation for 5 min in hot water (50 °C). For calibration, gallic acid (0.3 M) was applied as the chemical standard. TPC of the sample was expressed as gallic acid equivalent/g dry weight extract (GAE/g dw.). Blank was prepared to contain distilled water instead of extract.

### 4.5. Antibacterial Activity

The antimicrobial activity of EOs was assessed against two Gram-negative (*Escherichia coli* ATCC 25922 and *Salmonella enterica* ATCC 13076) and two Gram-positive (*Bacillus cereus* ATCC 10876 and *Staphylococcus aureus* ATCC 29213) reference bacterial strains with disc diffusion and broth microdilution methods based on the corresponding CLSI standards (CLSI M02 and CLSI M07 [53,54]). Few colonies from overnight agar plates were suspended in saline and the turbidity was adjusted to 0.5 McFarland and 50 µL of these suspensions were plated in the surface of Mueller–Hinton agar (Sigma-Aldrich). Blank paper discs (6 mm in diameter) were deposited on the plates inoculated with the bacterial suspensions and 5 µL oil was pipetted onto the discs. Gentamycin (10 µg/disc) was used as reference antibacterial agent. The plates were incubated at 35 ± 1 °C for 17 ± 1 h and the diameter of the inhibition zones were measured. All tests were performed in triplicate.

The minimum inhibitory concentration (MIC) of the tested oils were performed using the broth microdilution method. Two-fold serial dilutions of the essential oils were performed in Muller–Hinton broth (Sigma-Aldrich) supplemented with 1% DMSO to enhance the solubility of the oils. In this experiment the final concentration of oils ranged from 0.03% to 2%, *v*/*v*. An amount of 100 μL of the dilutions were applied to the wells of a 96-well plate in duplicate and inoculated with 100 μL of inoculum. To prepare the inoculum, the 0.5 McFarland suspensions were diluted in Muller–Hinton broth (supplemented with 1% DMSO) 150 fold, reaching a density of 5 × 10^5^ CFU/mL. At the same time, the growth control (no oils) and sterility test (no oils and no inoculum) were carried out. In the case of microdilution studies, gentamycin was used as a reference antibiotic at a concentration of 0.06–32 µg/mL. The prepared plates were incubated at 35 ± 1 °C for 18 ± 1 h. The MIC was determined as the lowest concentration of the tested oil with no growth of the bacteria measured spectrophotometrically using a microplate reader (OD600, Boeco BMR-100).

The OD600 data were used to calculate IC_50_ (half maximal inhibitory concentration) values, with the Quest Graph™ IC_50_ Calculator (AAT Bioquest 2023 [55]) and the minimum bactericidal concentration (MBC) was determined by sub-cultivation of 25 μL on Mueller–Hinton agar and was considered as the lowest concentration that killed 99.99% of the initial inoculum.

### 4.6. Statistical Evaluation

The statistical evaluation of the research results was conducted using R version 4.2. [56]. The dataset included the concentrations of the active compounds for eleven populations. During data pre-processing, missing data were removed, and numerical data were standardized by Z-score normalization. Linear Discriminant Analysis (LDA) was used to separate the populations, which allowed us to determine which active compounds contributed most to the separation between populations. In the LDA, the population variable was used as a dependent variable, while the concentration of each essential oil component was used as a predictor. The results of the data analysis were plotted on graphs (‘ggplot2’ package) [57], including the LDA axes (LD1 and LD2) showing the separation between populations. The performance of the LDA model was evaluated using a confusion matrix, which showed the agreement between actual and predicted classes. The effectiveness of the LDA was assessed using Wilks’ Lambda (λ). To determine significant differences between populations, a multivariate analysis of variance (MANOVA) was conducted to examine the total phenolic concentration, antioxidant capacity, and essential oil component concentrations across the eleven populations. Population differences were evaluated using Tukey’s post hoc test. The assumption of normality for the MANOVA was tested using the Shapiro–Wilk test, while the homogeneity of variances was assessed using Levene’s test. Partial eta-squared (η^2^) was calculated as a measure of effect size for the MANOVA results.

## 5. Conclusions

Six distinct mint species—peppermint (*M. × piperita*), spearmint (*M. spicata*, five chemotypes), horse mint (*M. longifolia*), mojito mint (*M. × villosa*), apple mint (*M. suaveolens*, two chemotypes), and bergamot mint (*M. aquatica* var. *citrata*)—were assessed for in vitro biological activity and chemical compositions. The essential oil (EO) concentration of the mint populations comprised 98 volatile components. The antibacterial efficacy of the oils was assessed against *Escherichia coli*, *Salmonella enterica*, *Bacillus cereus*, and *Staphylococcus aureus* by inhibition zone measurements, minimum inhibitory concentration (MIC), IC_50_, and minimum bactericidal concentration (MBC) values. The carvacrol–thymol chemotype of spearmint demonstrated the most robust antibacterial efficacy among the assessed samples. Bergamot mint showed limited antibacterial activity against Gram-negative bacteria, whereas oils high in dihydrocarvone exhibited the lowest efficacy against Gram-positive bacteria. For instance, none of the evaluated essential oils had bactericidal activity against *B. cereus* at the amounts used. These findings highlight the variation in the antibacterial activity of mint essential oils and indicate the impact of chemical composition on their bioactivity.

## Figures and Tables

**Table 1:** Essential oil composition of the analyzed mint samples (average percentage ± standard deviation).

Compound	RT	LRI	A. LRI	B1	B11	J14	B4	J7	B7	B12	J3	J17	J15	J4
**α-Thujene**	5.31	928	924	0.06 ± 0.02	1.78 ± 0.00	0.00 ± 0.00	0.00 ± 0.00	0.00 ± 0.00	0.05 ± 0.00	0.37 ± 0.00	0.00 ± 0.00	0.07 ± 0.02	0.00 ± 0.00	0.00 ± 0.00
**α-Pinene**	5.56	938	932	0.43 ± 0.12	0.63 ± 0.00	0.59 ± 0.38	0.43 ± 0.30	0.36 ± 0.05	0.60 ± 0.14	0.26 ± 0.23	0.21 ± 0.01	0.43 ± 0.13	0.48 ± 0.27	0.14 ± 0.04
**Sabinene**	6.52	976	969	0.72 ± 0.13	1.33 ± 0.00	1.09 ± 0.47	0.84 ± 0.40	0.45 ± 0.07	0.62 ± 0.07	0.39 ± 0.05	0.31 ± 0.01	0.42 ± 0.08	0.26 ± 0.10	0.39 ± 0.07
**ß-Pinene**	6.64	981	974	0.92 ± 0.17	1.35 ± 0.00	1.65 ± 0.72	1.52 ± 0.70	1.00 ± 0.08	0.90 ± 0.11	0.67 ± 0.06	0.47 ± 0.01	1.33 ± 0.10	0.63 ± 0.22	0.42 ± 0.07
**3-Octanol**	6.88	990	982	0.00 ± 0.00	0.85 ± 0.00	0.42 ± 0.02	0.52 ± 0.08	0.00 ± 0.00	0.21 ± 0.03	0.13 ± 0.02	0.12 ± 0.01	0.29 ± 0.08	0.13 ± 0.02	0.00 ± 0.00
**ß-Myrcene**	6.99	995	988	1.27 ± 0.10	1.71 ± 0.00	6.08 ± 0.03	6.54 ± 1.70	0.48 ± 0.10	1.90 ± 0.10	0.55 ± 0.10	0.20 ± 0.00	0.40 ± 0.10	0.36 ± 0.10	1.77 ± 0.10
**p-Cymene**	8.09	1026	1020	0.11 ± 0.00	12.97 ± 0.00	0.00 ± 0.00	0.00 ± 0.00	0.00 ± 0.00	0.00 ± 0.00	0.00 ± 0.00	0.00 ± 0.00	0.12 ± 0.03	0.00 ± 0.00	0.09 ± 0.01
**Limonene**	8.19	1029	1024	5.36 ± 0.10	0.00 ± 0.00	5.55 ± 0.10	6.02 ± 1.10	5.89 ± 0.20	18.81 ± 1.30	6.08 ± 0.00	4.12 ± 0.10	1.70 ± 0.10	1.87 ± 0.30	0.69 ± 0.10
**1,8-Cineole**	8.38	1034	1026	9.28 ± 0.80	21.36 ± 0.00	14.45 ± 0.40	15.50 ± 3.50	1.91 ± 0.00	4.72 ± 0.10	1.68 ± 0.20	5.18 ± 0.00	0.00 ± 0.00	0.13 ± 0.00	6.04 ± 0.20
**(Z)-Ocymene (*cis*-β-ocymene)**	8.50	1037	1032	0.21± 0.02	0.53 ± 0.00	0.74 ± 0.13	0.47 ± 0.11	0.39 ± 0.05	0.32 ± 0.01	0.35 ± 0.04	0.32 ± 0.03	0.26 ± 0.03	0.64 ± 0.06	1.01 ± 0.06
**(E)-Ocymene (*trans*-β-ocymene)**	8.85	1046	1044	0.10 ± 0.00	0.00 ± 0.00	0.07 ± 0.00	0.06 ± 0.00	0.00 ± 0.00	0.12 ± 0.01	0.00 ± 0.00	0.07 ± 0.01	0.00 ± 0.00	0.00 ± 0.00	0.69 ± 0.04
**γ-Terpinene**	9.20	1056	1054	0.72 ± 0.10	6.95 ± 0.00	0.11 ± 0.00	0.51 ± 0.30	0.00 ± 0.00	2.11 ± 0.10	0.00 ± 0.00	1.09 ± 0.10	0.00 ± 0.00	0.03 ± 0.10	0.19 ± 0.20
***Trans*-sabinene-hydrate**	9.73	1070	1098	10.29 ± 0.40	1.16 ± 0.00	0.00 ± 0.00	0.00 ± 0.00	0.00 ± 0.00	0.00 ± 0.00	0.00 ± 0.00	0.00 ± 0.00	9.91 ± 0.20	0.00 ± 0.00	0.22 ± 0.20
**α-Terpinolene**	10.29	1085	1086	0.17 ± 0.02	0.00 ± 0.00	0.00 ± 0.00	0.11 ± 0.01	0.00 ± 0.00	0.05 ± 0.00	0.00 ± 0.00	0.00 ± 0.00	0.00 ± 0.00	0.00 ± 0.00	0.20 ± 0.01
***Cis*-sabinene-hydrate**	10.72	1096	1065	0.33 ± 0.02	0.20 ± 0.00	0.00 ± 0.00	0.00 ± 0.00	0.00 ± 0.00	0.08 ± 0.02	0.00 ± 0.00	0.00 ± 0.00	0.25 ± 0.04	0.00 ± 0.00	0.00 ± 0.00
**Linalool**	10.76	1097	1095	0.00 ± 0.00	0.22 ± 0.00	0.27 ± 00.0	0.15 ± 0.10	0.00 ± 0.00	0.00 ± 0.00	0.00 ± 0.00	0.25 ± 0.00	0.00 ± 0.00	0.03 ± 0.10	23.12 ± 0.40
**Octen-3-yl acetate**	11.19	1108	1110	0.00 ± 0.00	0.00 ± 0.00	0.06 ± 0.00	0.00 ± 0.00	0.00 ± 0.00	0.00 ± 0.00	0.00 ± 0.00	0.00 ± 0.00	0.24 ± 0.01	0.00 ± 0.00	0.32 ± 0.11
**3-octanol-acetate**	11.85	1124	1120	0.00 ± 0.00	0.00 ± 0.00	0.00 ± 0.00	0.13 ± 0.01	0.00 ± 0.00	0.00 ± 0.00	0.00 ± 0.00	0.00 ± 0.00	0.00 ± 0.00	0.00 ± 0.00	0.78 ± 0.02
**Menthone**	13.27	1158	1148	0.00 ± 0.00	0.00 ± 0.00	0.21 ± 0.20	0.00 ± 0.00	0.00 ± 0.00	0.00 ± 0.00	0.20 ± 0.00	34.30 ± 2.90	0.00 ± 0.00	0.11 ± 0.10	0.67 ± 0.50
**Menthofuran**	13.28	1158	1159	0.00 ± 0.00	0.00 ± 0.00	0.00 ± 0.00	0.00 ± 0.00	0.00 ± 0.00	0.00 ± 0.00	0.00 ± 0.00	0.00 ± 0.00	0.00 ± 0.00	0.20 ± 0.08	0.00 ± 0.00
**Isoborneol**	14.26	1182	1179	0.00 ± 0.00	0.00 ± 0.00	0.00 ± 0.00	0.00 ± 0.00	0.31 ± 0.08	0.20 ± 0.02	0.00 ± 0.00	0.00 ± 0.00	0.46 ± 0.04	0.00 ± 0.00	0.00 ± 0.00
**α-Terpineol (*cis*-dehydro)**	13.49	1164	1143	0.48 ± 0.02	0.77 ± 0.00	0.77 ± 0.05	0.73 ± 0.14	0.00 ± 0.00	0.18 ± 0.01	0.00 ± 0.00	0.00 ± 0.00	0.00 ± 0.00	0.00 ± 0.00	0.00 ± 0.00
**Isomenthone**	13.67	1168	1293	0.00 ± 0.00	0.00 ± 0.00	0.00 ± 0.00	0.00 ± 0.00	0.00 ± 0.00	0.00 ± 0.00	0.00 ± 0.00	7.49 ± 0.10	0.00 ± 0.00	0.00 ± 0.00	0.27 ± 0.10
**Menthol**	13.80	1171	1167	0.00 ± 0.00	0.00 ± 0.00	0.17 ± 0.20	0.00 ± 0.00	0.00 ± 0.00	0.00 ± 0.00	0.00 ± 0.00	35.07 ± 2.60	0.00 ± 0.00	0.00 ± 0.00	0.25 ± 0.00
**Terpinene-4-ol**	13.96	1175	1174	1.94 ± 0.44	0.34 ± 0.00	0.51 ± 0.02	0.36 ± 0.06	0.00 ± 0.00	0.14 ± 0.03	0.00 ± 0.00	0.50 ± 0.05	0.75 ± 0.12	0.37 ± 0.00	4.34 ± 0.07
**Neo-iso-pulegol**	13.96	1175	1144	0.0 ± 0.00	0.00 ± 0.00	0.00 ± 0.00	0.00 ± 0.00	0.00 ± 0.00	0.00 ± 0.00	0.00 ± 0.00	0.00 ± 0.00	0.00 ± 0.00	5.63 ± 0.30	0.00 ± 0.00
**Isomenthol**	14.26	1182	1179	0.00 ± 0.00	0.00± 0.00	0.00 ± 0.00	0.00 ± 0.00	0.00 ± 0.00	0.00 ± 0.00	0.00 ± 0.00	0.36 ± 0.04	0.00 ± 0.00	0.00 ± 0.00	0.00 ± 0.00
***Cis*-dihydrocarvone**	14.74	1194	1191	0.46 ± 0.03	0.27 ± 0.00	0.14 ± 0.00	0.00 ± 0.00	32.63 ± 1.50	0.72 ± 0.10	43.05 ± 3.00	0.00 ± 0.00	0.00 ± 0.00	0.00 ± 0.00	0.00 ± 0.00
***Trans*-dihydrocarvone**	15.12	1203	1200	0.00 ± 0.00	0.10 ± 0.00	0.00 ± 0.00	0.00 ± 0.00	36.99 ± 2.00	0.00 ± 0.00	33.61 ± 2.60	0.00 ± 0.00	0.00 ± 0.00	0.00 ± 0.00	0.00 ± 0.00
***Trans*-carveol**	15.73	1217	1215	0.00 ± 0.00	0.00 ± 0.00	0.00 ± 0.00	0.00 ± 0.00	0.78 ± 0.09	0.00 ± 0.00	0.52 ± 0.13	0.00 ± 0.00	0.00 ± 0.00	0.00 ± 0.00	0.00 ± 0.00
**Nerol**	16.15	1227	1227	0.00 ± 0.00	0.13 ± 0.00	0.00 ± 0.00	0.00 ± 0.00	0.00 ± 0.00	0.00 ± 0.00	0.00 ± 0.00	0.00 ± 0.00	0.00 ± 0.00	0.00 ± 0.00	0.67 ± 0.02
***Cis*-carveol**	16.31	1231	1215	0.37± 0.04	0.00 ± 0.00	0.00 ± 0.00	0.00 ± 0.00	1.65 ± 0.08	0.00 ± 0.00	0.41 ± 0.35	0.00 ± 0.00	0.00 ± 0.00	0.00 ± 0.00	0.00 ± 0.00
**Pulegon**	16.50	1236	1233	0.00 ± 0.00	0.00 ± 0.00	0.84 ± 0.60	0.00 ± 0.00	0.00 ± 0.00	0.00 ± 0.00	0.00 ± 0.00	0.60 ± 0.00	0.69 ± 0.10	87.51 ± 2.10	0.00 ± 0.00
**L-carvone**	16.71	1241	249	59.97 ± 1.50	0.06 ± 0.00	0.09 ± 0.00	0.00 ± 0.00	0.27 ± 0.10	64.61 ± 1.60	0.72 ± 0.30	0.00 ± 0.00	0.05 ± 0.00	0.00 ±0.00	0.05 ± 0.10
**Piperitone**	17.08	1249	1249	0.00 ± 0.00	0.00 ± 0.00	0.00 ± 0.00	0.00± 0.00	0.00 ± 0.00	0.00 ± 0.00	0.00 ± 0.00	1.35 ± 0.06	0.00 ± 0.00	0.00 ± 0.00	0.00 ± 0.00
**Linalyl-acetate**	17.11	1250	1254	0.00 ± 0.00	0.10 ± 0.00	0.00 ± 0.00	0.00 ± 0.00	0.00 ± 0.00	0.00 ± 0.00	0.00 ± 0.00	0.00 ± 0.00	0.00 ± 0.00	0.00 ± 0.00	25.60 ± 0.30
***Cis*-piperitone-epoxide**	17.42	1257	1250	0.00 ± 0.00	0.00 ± 0.00	0.10 ± 0.10	0.00 ± 0.00	0.00 ± 0.00	0.00 ± 0.00	0.00 ± 0.00	0.00 ± 0.00	63.5 ± 1.00	0.20 ± 0.00	0.00 ± 0.00
***Trans*-piperitone-epoxide**	17.53	1260	1252	0.00 ± 0.00	0.00 ± 0.00	0.00 ± 0.00	0.00 ± 0.00	0.00 ± 0.00	0.00 ± 0.00	0.00 ± 0.00	0.00 ± 0.00	0.17 ± 0.03	0.00 ± 0.00	0.00 ± 0.00
**Thymol**	18.81	1290	1289	0.00 ± 0.00	13.00 ± 0.00	0.00 ± 0.00	0.00 ± 0.00	0.00 ± 0.00	0.00 ± 0.00	0.00 ± 0.00	0.00 ± 0.00	0.50 ± 0.00	0.00 ± 0.00	0.00 ± 0.00
**Menthyl-acetate**	18.84	1291	1294	0.00 ± 0.00	0.00 ± 0.00	0.00 ± 0.00	0.00 ± 0.00	0.00 ± 0.00	0.00 ± 0.00	0.00 ± 0.00	2.90 ± 0.40	0.00 ± 0.00	0.00 ± 0.00	0.00 ± 0.00
**Carvacrol**	19.20	1300	1370	0.00 ± 0.00	26.10 ± 0.00	0.00 ± 0.00	0.00 ± 0.00	0.00 ± 0.00	0.00 ± 0.00	0.00 ± 0.00	0.00 ± 0.00	0.00 ± 0.00	0.00 ± 0.00	0.00 ± 0.00
**Dihydro-carveol-acetate (iso)**	20.11	1324	1212	0.14 ± 0.03	0.00 ± 0.00	0.00 ± 0.00	0.00 ± 0.00	0.45 ± 0.09	0.00 ± 0.00	0.04 ± 0.08	0.00 ± 0.00	0.00 ± 0.00	0.00 ± 0.00	0.00 ± 0.00
***Trans*-piperitol-acetate**	20.91	1348	1343	0.00 ± 0.00	0.00 ± 0.00	0.00 ± 0.00	0.00 ± 0.00	2.00 ± 0.30	0.00 ± 0.00	0.00 ± 0.00	0.00 ± 0.00	0.00 ± 0.00	0.00 ± 0.00	0.00 ± 0.00
**Dihydro-carveol-acetate (neoiso)**	21.57	1364	1356	0.00 ± 0.00	0.00 ± 0.00	0.00 ± 0.00	0.00 ± 0.00	0.55 ± 0.11	0.00 ± 0.00	0.05 ± 0.09	0.00 ± 0.00	0.00 ± 0.00	0.00 ± 0.00	0.00 ± 0.00
**Neryl-acetate**	21.64	1366	1359	0.00 ± 0.00	0.00 ± 0.00	0.00 ± 0.00	0.00 ± 0.00	0.00 ± 0.00	0.00 ± 0.00	0.00 ± 0.00	0.00 ± 0.00	0.00 ± 0.00	0.00 ± 0.00	13.00 ± 0.00
**Linalyl-isobutanoate**	21.82	1371	1373	0.00 ± 0.00	0.00 ± 0.00	0.00 ± 0.00	0.00 ± 0.00	0.00 ± 0.00	0.00 ± 0.00	0.00 ± 0.00	0.00 ± 0.00	0.00 ± 0.00	0.00 ± 0.00	1.10 ± 0.04
**Piperitenone oxide**	21.97	1375	1366	0.00 ± 0.00	0.00 ± 0.00	52.13 ± 0.20	56.28 ± 5.30	0.00 ± 0.00	0.00 ± 0.00	0.00 ± 0.00	0.00 ± 0.00	5.45 ± 0.20	0.00 ± 0.00	0.00 ± 0.00
**ß-Bourbonene**	22.26	1383	1387	0.95 ± 0.10	0.00 ± 0.00	0.15 ± 0.01	0.24 ± 0.06	0.13 ± 0.04	0.32 ± 0.10	0.18 ± 0.15	0.28 ± 0.03	0.19 ± 0.01	0.00 ± 0.00	0.00 ± 0.00
**Geranyl-acetate**	22.43	1388	1379	0.00 ± 0.00	0.00 ± 0.00	0.00 ± 0.00	0.00 ± 0.00	0.00 ± 0.00	0.00 ± 0.00	0.00 ± 0.00	0.00 ± 0.00	0.00 ± 0.00	0.00 ± 0.00	2.09 ± 0.10
**ß-Elemene**	22.55	1391	1389	0.32 ± 0.03	0.00 ± 0.00	0.10 ± 0.01	0.24 ± 0.04	0.19 ± 0.05	0.05 ± 0.00	0.11 ± 0.10	0.14 ± 0.01	0.19 ± 0.01	0.00 ± 0.00	1.04 ± 0.01
***Cis*-jasmone**	23.04	1404	1392	0.00 ± 0.00	0.00 ± 0.00	0.41 ± 0.05	0.66 ± 0.10	0.00 ± 0.00	0.00 ± 0.00	0.00 ± 0.00	0.00 ± 0.00	0.00 ± 0.00	0.23 ± 0.09	0.30 ± 0.04
**ß-Caryophyllene**	23.68	1420	1417	1.14 ± 0.07	1.63 ± 0.00	4.99 ± 0.50	2.91 ± 0.30	4.88 ± 0.10	0.28 ± 0.10	3.44 ± 0.80	0.76 ± 0.00	3.68 ± 0.10	0.23 ± 0.10	2.70 ± 0.10
**α-Humulene**	25.07	1454	1452	0.00 ± 0.00	0.00 ± 0.00	0.19 ± 0.02	0.14 ± 0.03	0.18 ± 0.06	0.05 ± 0.00	0.10 ± 0.08	0.00 ± 0.00	0.44 ± 0.02	0.00 ± 0.00	0.22 ± 0.01
**ß-Farnesene**	25.27	1459	1454	0.09 ± 0.00	0.00 ± 0.00	0.00 ± 0.00	0.35 ± 0.16	0.00 ± 0.00	0.00 ± 0.00	0.00 ± 0.00	0.29 ± 0.03	0.32 ± 0.03	0.00 ± 0.00	0.10 ± 0.01
**Alloaromadendrene**	25.39	1462	1639	0.13 ± 0.01	0.00 ± 0.00	0.00 ± 0.00	0.00 ± 0.00	0.41 ± 0.12	0.00 ± 0.00	0.09 ± 0.16	0.00 ± 0.00	0.00 ± 0.00	0.00 ± 0.00	0.00 ± 0.00
**Germacrene-D**	26.18	1482	1480	0.52 ± 0.06	1.45 ± 0.00	1.50 ± 0.20	3.95 ± 0.60	4.02 ± 0.30	0.66 ± 0.20	4.21 ± 1.40	1.71 ± 0.10	5.55 ± 0.10	0.20 ± 0.10	2.57 ± 0.00
**Bicyclogermacrene**	26.81	1497	1500	0.18 ± 0.02	0.12 ± 0.00	0.00 ± 0.00	0.00 ± 0.00	0.74 ± 0.10	0.15 ± 0.05	0.61 ± 0.30	0.46 ± 0.03	0.00 ± 0.00	0.00 ± 0.00	0.20 ± 0.01
**δ-Cadinene**	27.80	1524	1513	0.00 ± 0.01	0.00 ± 0.00	0.98 ± 0.07	0.00 ± 0.00	0.59 ± 0.04	0.00 ± 0.00	0.00 ± 0.00	0.00 ± 0.00	0.00 ± 0.00	0.00 ± 0.00	0.00 ± 0.00
**Elemol**	28.86	1553	1548	0.00 ± 0.00	0.00 ± 0.00	0.00 ± 0.00	0.00 ± 0.00	0.00 ± 0.00	0.00 ± 0.00	0.00 ± 0.00	0.00 ± 0.00	0.00 ± 0.00	0.00 ± 0.00	6.32 ± 0.20
**ß-cadinene**	29.87	1581	1537	0.00 ± 0.00	0.19 ± 0.00	0.00 ± 0.00	0.00 ± 0.00	0.51 ± 0.09	0.00 ± 0.00	0.33 ± 0.29	0.17 ± 0.02	0.12 ± 0.00	0.00 ± 0.00	0.00 ± 0.00
**Spathulenol**	29.98	1584	1381	0.12 ± 0.01	0.00 ± 0.00	0.20 ± 0.01	0.00 ± 0.00	0.00 ± 0.00	0.13 ± 0.01	0.00 ± 0.00	0.00 ± 0.00	0.00 ± 0.00	0.29 ± 0.06	0.00 ± 0.00
**Caryophyllene-oxide**	30.20	1590	1582	0.10 ± 0.03	0.00 ± 0.00	0.78 ± 0.08	0.34 ± 0.10	0.11 ± 0.00	0.00 ± 0.00	0.00 ± 0.00	0.00 ± 0.00	0.12 ± 0.00	0.35 ± 0.07	0.11 ± 0.01
**Viridiflorol**	30.49	1598	1592	0.28 ± 0.01	0.42 ± 0.00	0.00 ± 0.00	0.00 ± 0.00	0.00 ± 0.00	0.00 ± 0.00	0.47 ± 0.21	0.48 ± 0.05	0.00 ± 0.00	0.00 ± 0.00	0.00 ± 0.00
**1,10-di-epi-Cubenol**	31.36	1621	1618	0.11 ± 0.00	0.00 ± 0.00	0.75 ± 0.06	0.00 ± 0.00	0.21 ± 0.06	0.00 ± 0.00	0.11 ± 0.10	0.00 ± 0.00	0.00 ± 0.00	0.00 ± 0.00	0.00 ± 0.00
**Tau-cadinol**	32.26	1644	1638	0.00 ± 0.00	0.00 ± 0.00	0.18 ± 0.02	0.00 ± 0.00	0.00 ± 0.00	0.00 ± 0.00	0.05 ± 0.09	0.00 ± 0.00	0.00 ± 0.00	0.00 ± 0.00	0.00 ± 0.00
**α-Cadinol**	32.77	1658	1652	0.11 ± 0.01	0.00 ± 0.00	0.52 ± 0.04	0.00 ± 0.00	0.220.05	0.00 ± 0.00	0.00 ± 0.00	0.00 ± 0.00	0.00 ± 0.00	0.00 ± 0.00	0.00 ± 0.00
Sum:				98.40	97.55	97.28	99.29	98.47	99.03	97.88	99.45	96.05	99.76	97.35

RT: retention time; LRI: linear retention index; A. LRI: Adams linear retention index; B1, B11, J14, B4, J7: *Mentha spicata;* B7: *Mentha × villosa*; B12: *Mentha longifolia*; J3: *Mentha × piperita*; J17, J15: *Mentha suaveolens*; J4: *Mentha aquatica.*

**Table 2:** The antibacterial activity (diameter of the inhibition zones and the MIC, IC_50_ and MBC values) of *Mentha* (M.) EOs against the four reference strains.

Species/Cultivar	Main Compounds in EO	Diameter of the Inhibition Zones (mm)				MIC (*v*/*v*%)			
		*E. coli*	*S. enterica*	*B. cereus*	*S. aureus*	*E. coli*	*S. enterica*	*B. cereus*	*S. aureus*
*M. spicata* B1	L-carvone	9.67 ± 0.58	11.00 ± 0.00	10.67 ± 0.58	21.67 ± 2.08	0.50	0.50	0.50	0.50
*M. spicata* B11	carvacrol, thymol	20.00 ± 1.00	20.67 ± 1.53	20.67 ± 2.52	22.33 ± 1.15	0.06	0.06	0.06	0.06
*M. spicata* J14	piperitenone oxid	10.00 ± 0.00	10.00 ± 1.00	14.33 ± 0.58	10.33 ± 0.58	0.50	0.5	0.25	0.25
*M. spicata* B4	piperitenone oxid	10.33 ± 0.58	9.00 ± 0.00	14.33 ± 0.58	10.33 ± 0.58	1.00	1.00	0.50	0.50
*M. spicata* J7	dihydrocarvones	8.00 ± 0.00	7.00 ± 0.00	9.67 ± 0.58	10.00 ± 0.00	0.50	1.00	1.00	1.00
*M. villosa* B7	L-carvone	10.00 ± 1.00	9.67 ± 1.53	10.67 ± 0.58	18.67 ± 1.53	0.25	0.50	0.50	0.50
*M. longifolia* B12	dihydrocarvones	9.00 ± 0.00	8.00 ± 1.00	9.00 ± 0.00	9.33 ± 0.58	0.50	1.00	2.00	1.00
*Mentha x piperita* J3	menthol, menthone	10.67 ± 0.58	9.00 ± 0.00	10.00 ± 2.00	16.33 ± 1.53	0.50	0.50	0.13	0.13
*M. suaveolens* J17	*cis*-piperitone-epoxid	8.67 ± 0.58	8.33 ± 0.58	14.00 ± 1.73	12.00 ± 1.00	0.50	0.50	0.13	0.25
*M. suaveolens* J15	pulegone	11.00 ± 1.73	12.00 ± 1.00	12.67 ± 1.53	12.00 ± 1.00	0.50	0.50	0.50	0.25
*M. aquatica* J4	linalool, linalyl-acetate	8.67 ± 0.58	7.67 ± 0.58	21.00 ± 3.61	20.67 ± 2.08	2.00	>2.00	0.50	0.50
Gentamycin ^1^	-	22.33 ± 0.58	22.33 ± 0.58	20.00 ± 1.00	19.67 ± 0.58	1.00	1.00	1.00	0.25
Species/Cultivar	Main compounds in EO	IC 50 (*v*/*v*%)				MBC (*v*/*v*%)			
		*E. coli*	*S. enterica*	*B. cereus*	*S. aureus*	*E. coli*	*S. enterica*	*B. cereus*	*S. aureus*
*M. spicata* B1	L-carvone	0.12 ± 0.03	0.10 ± 0.03	0.15 ± 0.02	0.08 ± 0.02	0.50	0.50	>2.00	>2.00
*M. spicata* B11	carvacrol, thymol	0.03 ± 0.01	0.03 ± 0.01	0.01 ± 0.01	0.02 ± 0.01	0.06	0.06	>2.00	0.06
*M. spicata* J14	piperitenone oxid	0.13 ± 0.02	0.09 ± 0.01	0.09 ± 0.01	0.12 ± 0.01	0.50	0.50	>2.00	1.00
*M. spicata* B4	piperitenone oxid	0.24 ± 0.01	0.22 ± 0.01	0.19 ± 0.01	0.27 ± 0.01	1.00	2.00	>2.00	2.00
*M. spicata* J7	dihydrocarvones	0.16 ± 0.02	0.15 ± 0.01	0.26 ± 0.03	0.37 ± 0.05	0.50	1.00	>2.00	>2.00
*M. villosa* B7	L-carvone	0.10 ± 0.01	0.09 ± 0.02	0.19 ± 0.05	0.06 ± 0.01	0.50	0.50	>2.00	>2.00
*M. longifolia* B12	dihydrocarvones	0.17 ± 0.05	0.15 ± 0.01	0.22 ± 0.05	0.36 ± 0.03	0.50	1.00	>2.00	>2.00
*Mentha x piperita* J3	menthol, menthone	0.09 ± 0.01	0.06 ± 0.02	0.07 ± 0.01	0.08 ± 0.01	0.50	1.00	>2.00	0.50
*M. suaveolens* J17	*cis*-piperitone-epoxid	0.10 ± 0.02	0.12 ± 0.01	0.06 ± 0.01	0.08 ± 0.01	0.50	0.50	>2.00	2.00
*M. suaveolens* J15	pulegone	0.12 ± 0.02	0.07 ± 0.01	0.14 ± 0.01	0.09 ± 0.01	0.50	0.50	>2.00	>2.00
*M. aquatica* J4	linalool, linalyl-acetate	0.25 ± 0.02	0.11 ± 0.02	0.12 ± 0.03	0.14 ± 0.01	2.00	>2.00	>2.00	>2.00
Gentamycin ^1^	-	0.26 ± 0.02	0.31 ± 0.02	0.60 ± 0.02	0.15 ± 0.01	1.00	1.00	8.00	0.50

^1^: In the case of disc diffusion, we used 6 mm diameter discs containing 10 µg of gentamycin. For MIC, IC_50_, and MBC, the concentration of gentamycin is given in mg/L.

## Data Availability

The original contributions presented in this study are included in this article. Further inquiries can be directed to the corresponding author.
